# Comparative Efficacy and Safety of Corticosteroids vs. No Corticosteroids in Septic Shock Intensive Care Unit Patients: A Systematic Review and Meta-Analysis

**DOI:** 10.14740/jocmr6631

**Published:** 2026-10-23

**Authors:** Aymen Ali Alqurain, Ali Abdullah Shahmurad Albalushi, Amr Korani Soltan Abdeltawab, Khadeijah Ahmed Almarshoodi, Fahd Alrumaih, Wateen Mohammed Almutairi, Bayan Abdullah Alnasser, Omar Ibrahim Alsaleem, Abdullah Ibrahim Aldhobaib, Thamer Ibrahim Alshridha, Aryam Ahmad Alomaireeni, Nader Faleh Alenzi, Rawabi Mohammed Alwashmi, Abdullah Mohammed Muhunna Alwashmi, Khayal Abaalkhayl

**Affiliations:** a Department of Clinical Practice, Faculty of Pharmacy, Northern Border University, Rafha, Saudi Arabia; b Pediatric Emergency Medicine, Ministry of Health, Sohar Hospital, Sohar, Oman; c Department of Intensive Care Unit, Althaghr Hospital, Jeddah, Saudi Arabia; d Department of Pharmacy Practice and therapeutics, College of Pharmacy, University of Sharjah (UOS), Fujairah, United Arab Emirates; e Department of Emergency Medicine, College of Medicine, Prince Sattam Bin Abdulaziz University, Riyadh, Saudi Arabia; f College of Pharmacy, Qassim University, Al-Qassim, Saudi Arabia; g College of Pharmacy, Buraydah Colleges, Al-Qassim, Saudi Arabia; h College of Medicine, Qassim University, Al-Qassim, Saudi Arabia

**Keywords:** Septic shock, Corticosteroids, Hydrocortisone, Mortality, Meta-analysis, Intensive care, Vasopressor support

## Abstract

**Background:**

Septic shock carries a high intensive care unit (ICU) mortality. The role of adjunctive corticosteroid therapy is controversial, with conflicting signals from trial-level meta-analyses, individual patient data syntheses, and large randomized trials. This review aimed to assess the comparative efficacy and safety of systemic corticosteroids versus no corticosteroids in adult ICU patients with septic shock.

**Methods:**

Following the PRISMA 2020 guidelines, MEDLINE, Embase, CENTRAL, and Scopus were searched from inception, supplemented by trial registry and gray literature searches, for randomized trials of systemic corticosteroids versus placebo or standard care in adults with septic shock. Pooling used a random-effects model with restricted maximum likelihood estimation and Hartung–Knapp–Sidik–Jonkman adjustment. The Risk of Bias 2 and Grading of Recommendations, Assessment, Development, and Evaluation (GRADE) frameworks were used for the methodological appraisal. Heterogeneity was quantified using I^2^, τ^2^, and 95% prediction intervals. Publication bias was assessed using contour-enhanced funnel plots, Egger’s regression test, and the Duval–Tweedie trim-and-fill method.

**Results:**

Eleven randomized controlled trials (RCTs) (n = 6,332; 1,929 deaths) were included. Corticosteroids reduced 28-day all-cause mortality by 8.7% relative to control (pooled risk ratio (RR) 0.91, 95% confidence interval (CI) 0.86–0.97; P = 0.008; I^2^ = 0%; prediction interval 0.84–0.99), corresponding to 27 fewer deaths per 1,000 (10–44 fewer). Time to shock reversal was shortened by 1.25 days (95% CI −2.09 to −0.41), with the standardized mean difference (SMD) sensitivity analysis confirming a robust effect (SMD −0.41, 95% CI −0.51 to −0.32). Shock reversal incidence (RR 1.08, 0.99–1.19) and ICU length of stay (LOS) (mean difference (MD) −2.07 days, −6.50 to 2.36) favored corticosteroids without reaching significance. Hyperglycemia occurred more frequently in the corticosteroid arm of every contributing trial. Egger’s test (P = 0.972) and trim-and-fill imputation provided no evidence of small-study effects. GRADE certainty was moderate for mortality and shock reversal incidence, high for time to shock reversal, and low for ICU LOS and hyperglycemia.

**Conclusion:**

Corticosteroid therapy in adults with septic shock was associated with a reduction in 28-day mortality and with accelerated shock reversal, with no evidence of publication bias. The direction of effect was consistent across pre-specified subgroups but was offset by a consistent excess of hyperglycemia, reinforcing the need for vigilant glucose surveillance during corticosteroid administration.

## Introduction

Septic shock is the most severe and life-threatening manifestation of sepsis, characterized by circulatory, cellular, and metabolic abnormalities that lead to multi-organ dysfunction and high mortality rates [1]. Standard management relies on prompt source control, broad-spectrum antimicrobial therapy, and aggressive hemodynamic resuscitation using intravenous fluids and vasopressors. Despite investigations and advancements in critical care, the routine administration of adjunctive corticosteroids to stabilize these patients remains controversial [1].

The pathophysiological rationale for utilizing systemic corticosteroids in septic shock is based on their ability to mitigate the dysregulated and exaggerated systemic inflammatory response, as well as to treat critical illness-related corticosteroid insufficiency (CIRCI) [2]. Physiologic or low-dose corticosteroids, such as hydrocortisone, have been investigated for their potential to restore vasomotor tone, reduce the duration of vasopressor dependency (e.g., norepinephrine), and alter immunoinflammatory profiles [3]. Furthermore, potential synergistic interactions between corticosteroids and other vasoactive agents, such as vasopressin, have been explored to optimize hemodynamic stabilization in refractory cases [4].

However, the clinical evidence regarding the definitive survival benefits of corticosteroids remains highly polarized. While some propensity-matched cohort studies and trial-level meta-analyses suggest that corticosteroids may facilitate earlier shock reversal and confer a potential survival advantage [5, 6], patient-level meta-analyses and trial sequential analyses have failed to demonstrate a definitive, reproducible reduction in short-term or long-term mortality [7, 8]. The overall cost-effectiveness and true impact of corticosteroid therapy on long-term health-related quality of life remain questionable, leaving clinicians without a clear consensus [9].

Therapeutics are convoluted by novel fixed-combination therapies and the recognition of highly specific patient phenotypes. Recent randomized controlled trials (RCTs) evaluating the early administration of hydrocortisone alongside ascorbic acid (vitamin C) and thiamine (HAT therapy) have yielded disappointing results, failing to demonstrate substantial improvements in mortality or organ dysfunction compared to standard care or placebo [10, 11]. Similarly, the routine addition of mineralocorticoids (fludrocortisone) to hydrocortisone regimens has shown mixed effects on overall mortality, warranting further systematic syntheses [12]. Furthermore, the safety profile of corticosteroids in highly vulnerable subpopulations, notably immunocompromised patients, is a significant clinical concern. Observational cohort data indicate the potential risks of adverse events, such as secondary infections and hyperglycemia, which may offset any short-term hemodynamic benefits in these critically ill cohorts [13].

Given the persistent clinical equipoise, heterogeneity of reported outcomes in recent high-profile trials, and need to isolate the independent effect of corticosteroids from complex combination regimens, an updated and rigorous evaluation is imperative. Therefore, this systematic review and meta-analysis aimed to assess the comparative efficacy and safety of corticosteroids versus no corticosteroids in adult intensive care unit (ICU) patients with septic shock.

## Materials and Methods

### Protocol registration and reporting standards

This systematic review and meta-analysis was conducted in accordance with the Preferred Reporting Items for Systematic Reviews and Meta-Analyses (PRISMA) 2020 guidelines (Supplementary Material 1, jocmr.elmerjournals.com) [14]. The overarching methodology and statistical analysis plan were prospectively registered in the International Prospective Register of Systematic Reviews (PROSPERO; registration number: CRD420261305337) to ensure methodological transparency and to mitigate selective reporting bias.

### Ethical compliance

As study involved a secondary analysis of published literature, ethical approval or patient consent was not required.

### Search strategy and information sources

A systematic literature search was performed across major electronic databases, including MEDLINE (via PubMed), Embase, the Cochrane Central Register of Controlled Trials (CENTRAL), and Scopus, from inception to the present. The search syntax was constructed using a combination of controlled vocabulary (e.g., MeSH terms, Emtree) AND, OR, NOT free-text keywords with appropriate Boolean operators (AND, OR, NOT) and wildcards. The search terms captured domains related to “septic shock,” “corticosteroids,” “hydrocortisone,” “dexamethasone,” and “randomized controlled trials.” To mitigate publication bias, gray literature and ongoing trials were screened using ClinicalTrials.gov and the WHO International Clinical Trials Registry Platform (ICTRP). No language or publication date restrictions were imposed. Backward and forward citation tracking (“snowballing”) of the included studies and relevant review articles was also performed.

### Eligibility criteria

Studies were selected based on the Population, Intervention, Comparator, Outcomes, and Study design (PICOS) framework. The population included adult patients (≥ 18 years) admitted to an ICU with a confirmed diagnosis of septic shock, defined by the necessity for vasopressor support to maintain a mean arterial pressure (MAP) ≥ 65 mm Hg and evidence of tissue hypoperfusion (e.g., elevated serum lactate), consistent with the Sepsis-3 consensus or equivalent preceding definitions.

The intervention was the systemic administration of corticosteroids (e.g., hydrocortisone, dexamethasone, methylprednisolone) at any dose (low/physiologic vs. high dose), timing, or duration, either alone or in combination with mineralocorticoids (e.g., fludrocortisone). The comparator was a placebo or standard care without adjunctive corticosteroid therapy.

The primary outcomes were 28-day all-cause mortality and rate/time to shock reversal, while the secondary endpoints included ICU and hospital length of stay (LOS), duration of mechanical ventilation, vasopressor-free days, and treatment-emergent adverse events (e.g., hyperglycemia, hypernatremia, secondary healthcare-associated infections, and gastrointestinal hemorrhage). Only RCTs were included, while observational studies, crossover trials, non-randomized designs, and pediatric cohorts were excluded.

### Study selection and data extraction

Two investigators independently screened the titles and abstracts of the retrieved records, followed by a full-text evaluation of the potentially eligible articles. Inter-rater reliability (IRR) for study inclusion was quantified using Cohen’s kappa coefficient (κ), with κ > 0.80 considered indicative of strong agreement [15]. Cohen’s κ was computed on all full-text reports assessed for eligibility. Discrepancies were resolved through adjudication by a third reviewer. Data extraction was independently performed using pre-piloted standardized forms, capturing study characteristics, patient demographics, illness severity scores (Acute Physiology and Chronic Health Evaluation II (APACHE II)/Sequential Organ Failure Assessment (SOFA)), intervention details, and specific outcome metrics.

### Quality assessment and risk of bias

The methodological quality of the included RCTs was appraised by two independent reviewers using the revised Cochrane Risk of Bias 2 (RoB 2) tool [16]. The studies were evaluated across five domains: randomization process, deviations from intended interventions, missing outcome data, measurement of the outcome, and selection of the reported result. Each trial was graded as having a “low,” “some concerns,” or “high” risk of bias.

### Statistical analysis and data synthesis

All statistical analyses were programmed and executed using R software (version 4.5.2; R Foundation for Statistical Computing, Vienna, Austria) utilizing the meta and metafor packages [17].

#### Effect size measures

For dichotomous outcomes (e.g., mortality, adverse events, and shock reversal incidence), effect sizes were pooled as risk ratios (RRs) with 95% confidence intervals (CIs). Continuous outcomes (e.g., LOS, vasopressor-free days, SOFA score changes) were analyzed using mean differences (MDs) or standardized mean differences (SMDs), depending on the uniformity of the measurement scales across trials.

#### Pooling models and estimators

Anticipating clinical and methodological heterogeneity, a random-effects (RE) model was employed for all primary syntheses. To provide error estimation, the restricted maximum likelihood (REML) estimator was applied, coupled with the Hartung–Knapp–Sidik–Jonkman (HKSJ) adjustment, to calculate the pooled effect sizes and 95% CIs [18].

#### Heterogeneity assessment and exploration

Statistical heterogeneity was quantified using Cochran’s Q test (significance defined as P < 0.10) and the I^2^ statistic, where I^2^ > 50% indicated substantial heterogeneity. The between-study variance was estimated using the τ^2^ (tau-squared) statistic, and a 95% prediction interval (PI) was calculated to project the expected range of true effects in future clinical settings [19]. To explore the sources of heterogeneity, *a priori* subgroup analyses (e.g., early vs. late initiation, low vs. high severity of illness, and immunocompetent vs. immunocompromised) and hierarchical meta-regression models (e.g., dose-response relationships) were performed [20]. Leave-one-out sensitivity analyses were conducted to evaluate the robustness of pooled estimates and identify highly influential outlier studies.

#### Publication bias

Small-study effects and publication bias were visually assessed using contour-enhanced funnel plots. Statistical detection was performed using Egger’s regression test (threshold P < 0.10). If asymmetry was detected, the Duval and Tweedie Trim-and-Fill method was applied to estimate the impact of potentially unpublished negative trials on the overall pooled effect [21, 22].

### Certainty of evidence

The overall certainty and quality of the body of evidence for each primary and key secondary outcome were evaluated using the Grading of Recommendations, Assessment, Development, and Evaluation (GRADE) framework [23]. Evidence certainty was downgraded based on the risk of bias, inconsistency, indirectness, imprecision, and publication bias.

## Results

### Study selection and IRR

A systematic search of MEDLINE, Embase, the Cochrane Central Register of Controlled Trials, and Scopus, supplemented by hand-searching of ClinicalTrials.gov and the WHO ICTRP, retrieved 3,186 records. After the removal of 331 duplicates, 2,855 unique records progressed to title and abstract screening, of which 1,850 were excluded as not meeting the eligibility criteria. Of the 1,005 reports sought for retrieval, 951 were excluded after full-text screening; the remaining 54 were assessed against the full PICOS criteria. Forty-three reports were subsequently excluded: 31 for not addressing the population of interest or reporting only short ICU admissions, 11 for containing insufficient data for quantitative synthesis, and one as a duplicate publication (a republication of the Gordon 2014 VANISH pilot). Eleven RCTs [24–34] enrolling 6,332 critically ill adults with septic shock were retained for the meta-analysis (Fig. 1). The inter-rater agreement between the two independent reviewers during eligibility adjudication was excellent, with Cohen’s κ = 0.89 (z = 4.02, P < 0.001), exceeding the prespecified threshold (κ > 0.80) for strong concordance.

**Figure 1:**
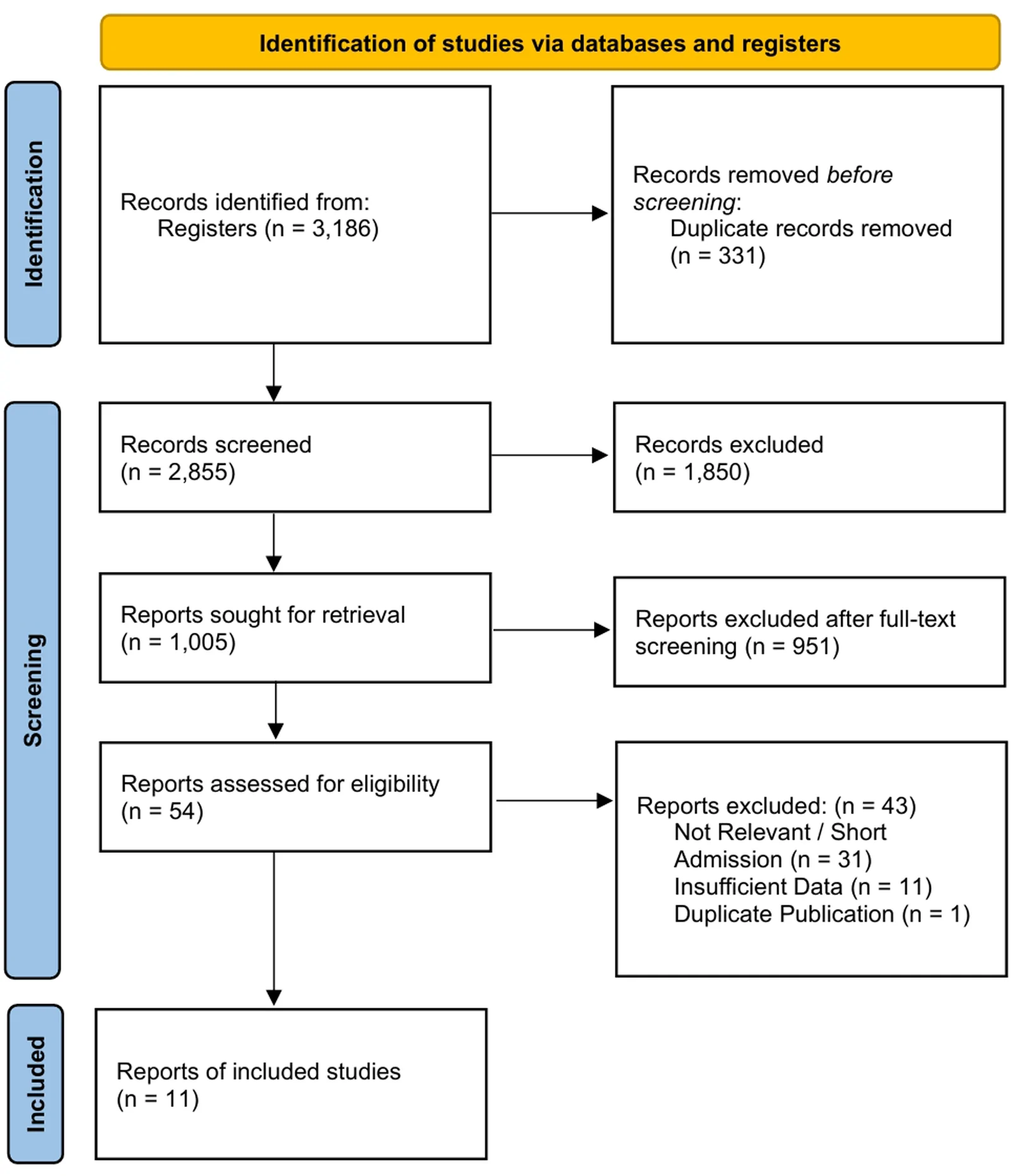
PRISMA 2020 flow diagram.

### Characteristics of included studies

The 11 included trials were conducted across nine countries spanning four continents and were published between 2002 and 2025 (baseline characteristics are detailed in Table 1). The sample sizes ranged from 29 [27] to 3,713 [34], with three large multicenter trials, APROCCHSS [26], ADRENAL [34], and CORTICUS [32], contributing 5,453 randomized participants and 86.1% of the pooled mortality cohort. Adjunctive corticosteroid regimens were heterogeneous in form but conserved in intent: nine trials evaluated hydrocortisone (most commonly 200 mg/day, administered as 50 mg every 6 h or as a continuous infusion, for 5–7 days); two trials [25, 26] combined hydrocortisone with enteral fludrocortisone (50 µg/day); and one trial [27] used pulsed dexamethasone (0.2 mg/kg every 36 h). Two trials enrolled patients with early septic shock, Lv 2017 within 6 h of onset [29] and Oppert 2005 in the early hyperdynamic phase [31], while three studies enrolled special populations (geriatric [24], cirrhotic [30], and refractory shock [33]); the remainder enrolled general adult ICU populations. Vasopressin co-treatment was protocolized in one trial [28].

**Table 1:** Baseline Characteristics of Included Studies

Study	Author, year	Country	Population/setting	Intervention (drug, dose, duration)	Comparator	Total N (intervention/control)
[24]	Agarwal, 2022	India	Geriatric patients (> 60 years) with septic shock in ICU	Hydrocortisone: 200 mg/day IV in four divided doses, tapered after shock reversal	Standard therapy	120 (61/59)
[25]	Annane, 2002	France	Adults with septic shock (ACTH non-responders & responders)	Hydrocortisone 50 mg IV q6h + fludrocortisone 50 µg/day enteral for 7 days	Placebo	299 (150/149)
[26]	Annane, 2018	France	Adults with septic shock (APROCCHSS trial)	Hydrocortisone 50 mg IV q6h + fludrocortisone 50 µg/day enteral for 7 days	Placebo	1241 (614/627)
[27]	Cicarelli, 2007	Brazil	Adults with septic shock	Dexamethasone: 0.2 mg/kg IV, three doses at 36 h intervals	Placebo	29 (14/15)
[28]	Gordon, 2014	UK	Adults with septic shock (VANISH pilot)	Vasopressin + hydrocortisone 50 mg IV q6h	Vasopressin + placebo	61 (31/30)
[29]	Lv, 2017	China	Adults with septic shock (within 6 h of onset)	Hydrocortisone: 200 mg/day continuous infusion for 6 days, then tapered	Placebo	118 (58/60)
[30]	Meersseman, 2025	Europe	Cirrhotic patients with septic shock	Hydrocortisone: 100 mg bolus + 200 mg/day continuous infusion for ≥ 3 days	Placebo	83 (40/43)
[31]	Oppert, 2005	Germany	Adults with early hyperdynamic septic shock	Hydrocortisone: 50 mg bolus + 0.18 mg/kg/h continuous infusion, then tapered	Placebo	41 (18/23)
[32]	Sprung, 2008	Int'l	Adults with septic shock (CORTICUS trial)	Hydrocortisone: 50 mg IV q6h for 5 days, then tapered	Placebo	499 (251/248)
[33]	Talebi Doluee, 2018	Iran	Adults with refractory septic shock	Hydrocortisone: 50 mg IV q6h for 7 days	Placebo	160 (80/80)
[34]	Venkatesh, 2018	Int'l	Adults with septic shock on mechanical ventilation (ADRENAL)	Hydrocortisone: 200 mg/day continuous infusion for 7 days	Placebo	3,713 (1,853/1,860)^a^

^a^ADRENAL [34] randomized 1,853 intervention/1,860 control (total 3,713); 1,841/1,840 were analyzed for 28-day mortality. ACTH: adrenocorticotropic hormone; ICU: intensive care unit; IV: intravenous.

### Risk of bias of included trials

The study-level RoB 2 judgements are summarized as a traffic-light plot in Figure 2 and as a weighted domain summary in Figure 3. Five trials [25, 26, 30, 32, 34] were judged to have a low overall risk of bias and contributed 90.9% of the meta-analytic weight for the primary mortality outcome. Four trials were rated as having some concerns: Cicarelli [27] for selective reporting (Domain 5), Lv [29] for missing outcome data (Domain 3), and Oppert [31] together with Talebi Doluee [33] for inadequate description of allocation concealment (Domain 1; sealed envelopes without third-party central randomization). Two trials [24, 28] were judged to have a high overall risk of bias, driven by their open-label or single-blind design and the consequent risk of deviations from the intended interventions (Domain 2). Across domains, 100% of trials were at low risk for outcome measurement (Domain 4), 91% for missing outcome data (Domain 3), 82% for randomization process (Domain 1), 91% for selective reporting (Domain 5), and 82% for deviations from intended interventions (Domain 2).

**Figure 2:**
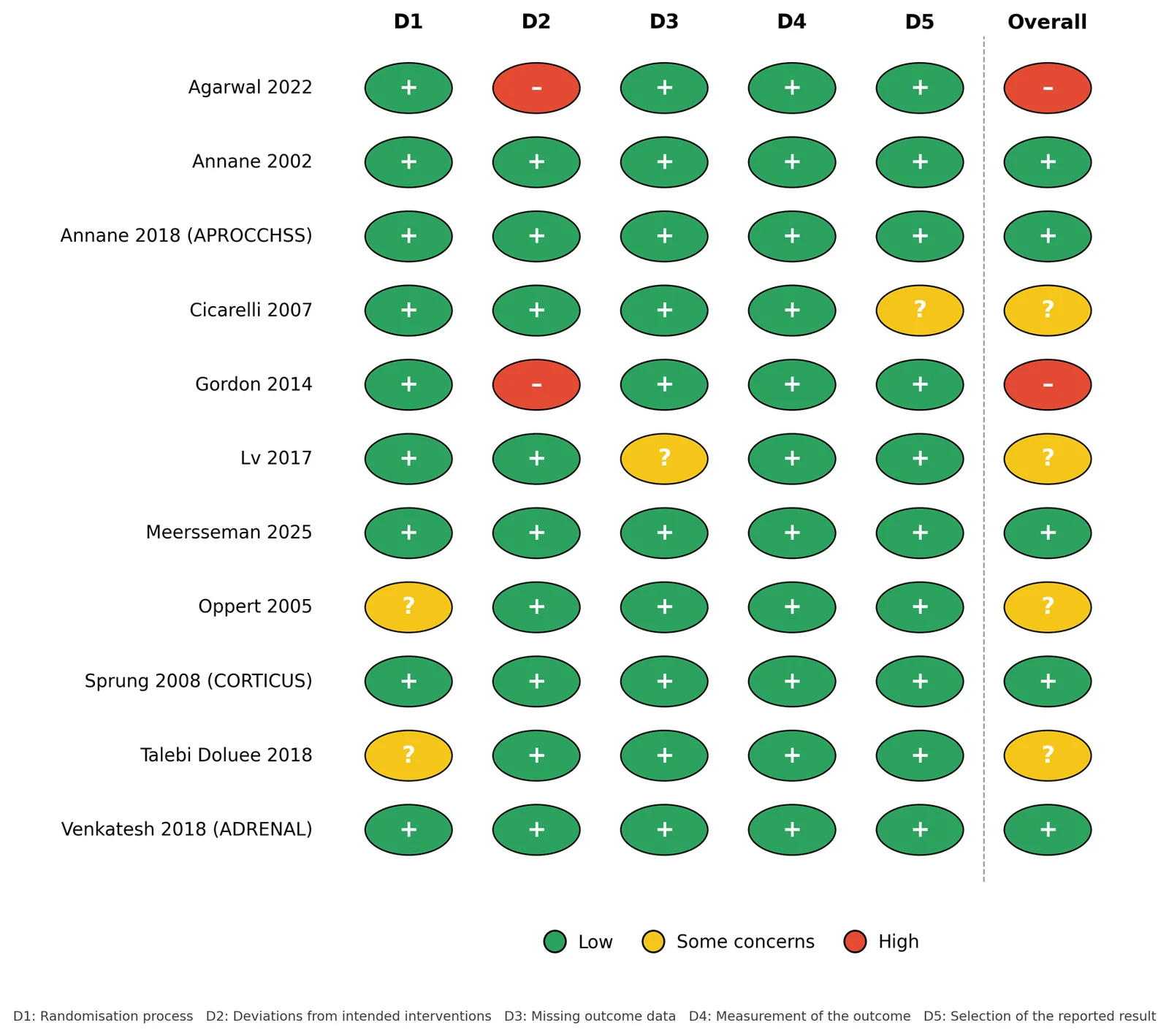
Risk of Bias 2 (RoB 2) traffic-light plot displaying per-domain (D1–D5) and overall judgements for each of the 11 included trials. Green (+) denotes “low risk of bias,” amber (?) denotes “some concerns,” and red (–) denotes “high risk of bias.”

**Figure 3:**
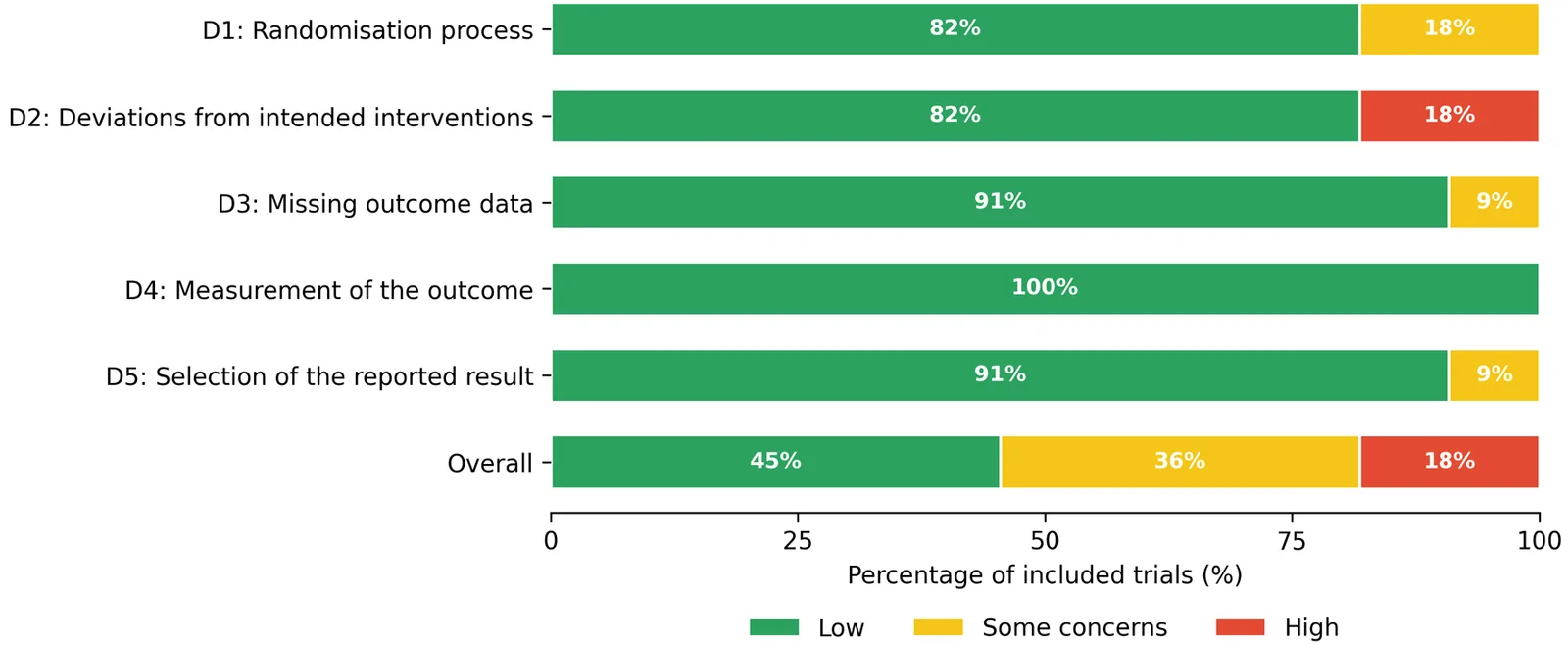
Weighted summary of Risk of Bias 2 judgements across the five domains and the overall rating. Each horizontal bar shows the percentage of included trials adjudicated as “low,” “some concerns,” or “high” in the corresponding domain.

### Primary outcome: 28-day all-cause mortality

All 11 trials reported 28-day all-cause mortality (n = 6,332 analyzable participants; 1,929 deaths). Compared with placebo or standard care, adjunctive corticosteroid therapy was associated with a statistically significant 8.7% relative reduction in 28-day mortality: pooled RR = 0.91 (95% CI 0.86–0.97; t_10_ = −3.29; P = 0.008) under the RE REML estimator with HKSJ adjustment (Fig. 4). Between-study heterogeneity was negligible (τ^2^ = 0; I^2^ = 0.0%, 95% CI 0.0–60.2%; Cochran’s Q = 5.91, df = 10, P = 0.822), and the 95% PI, 0.84–0.99, likewise excluded the null, supporting the generalizability of the survival benefit to plausible future settings drawn from the same population. The dominant contributors to the pooled estimate were ADRENAL (35.7% weight; RR 0.92, 95% CI 0.81–1.03), APROCCHSS (22.4%; RR 0.87, 0.75–1.01), and Annane 2002 (13.1%; RR 0.90, 0.74–1.09), all of which directionally favored corticosteroids.

**Figure 4:**
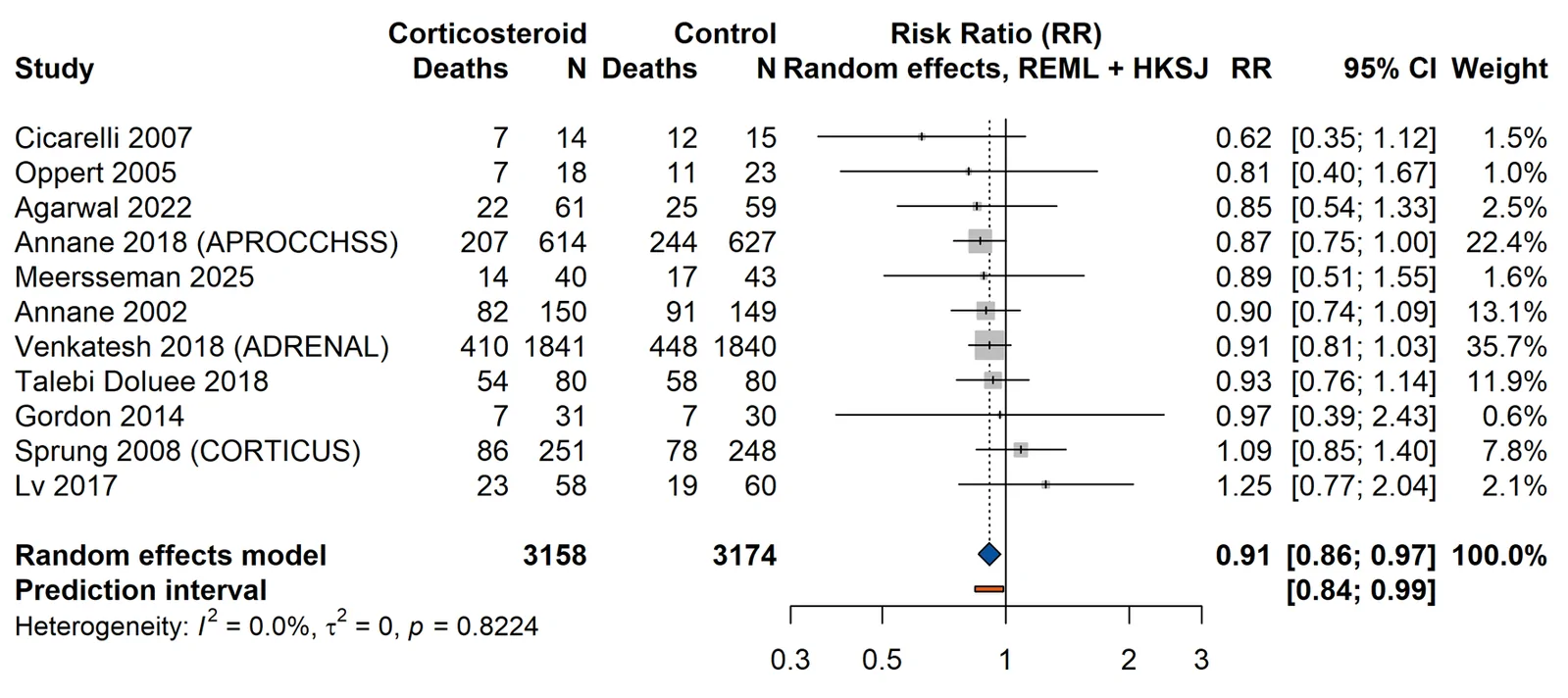
Forest plot of pooled risk ratios for 28-day all-cause mortality (random-effects model with REML estimator and Hartung–Knapp–Sidik–Jonkman adjustment). Square markers represent individual trial estimates with their 95% confidence intervals, sized by inverse-variance weight; the diamond denotes the pooled estimate; the orange band represents the 95% prediction interval. REML: restricted maximum likelihood.

The leave-one-out sensitivity analysis confirmed the robustness of the pooled effect: omission of any single trial yielded a re-pooled RR between 0.90 and 0.93, with statistical significance preserved in all 11 iterations and only marginal attenuation upon exclusion of APROCCHSS (P = 0.039) or ADRENAL (P = 0.033) (Fig. 5). No individual trial was identified as a heterogeneity-driving outlier in this study.

**Figure 5:**
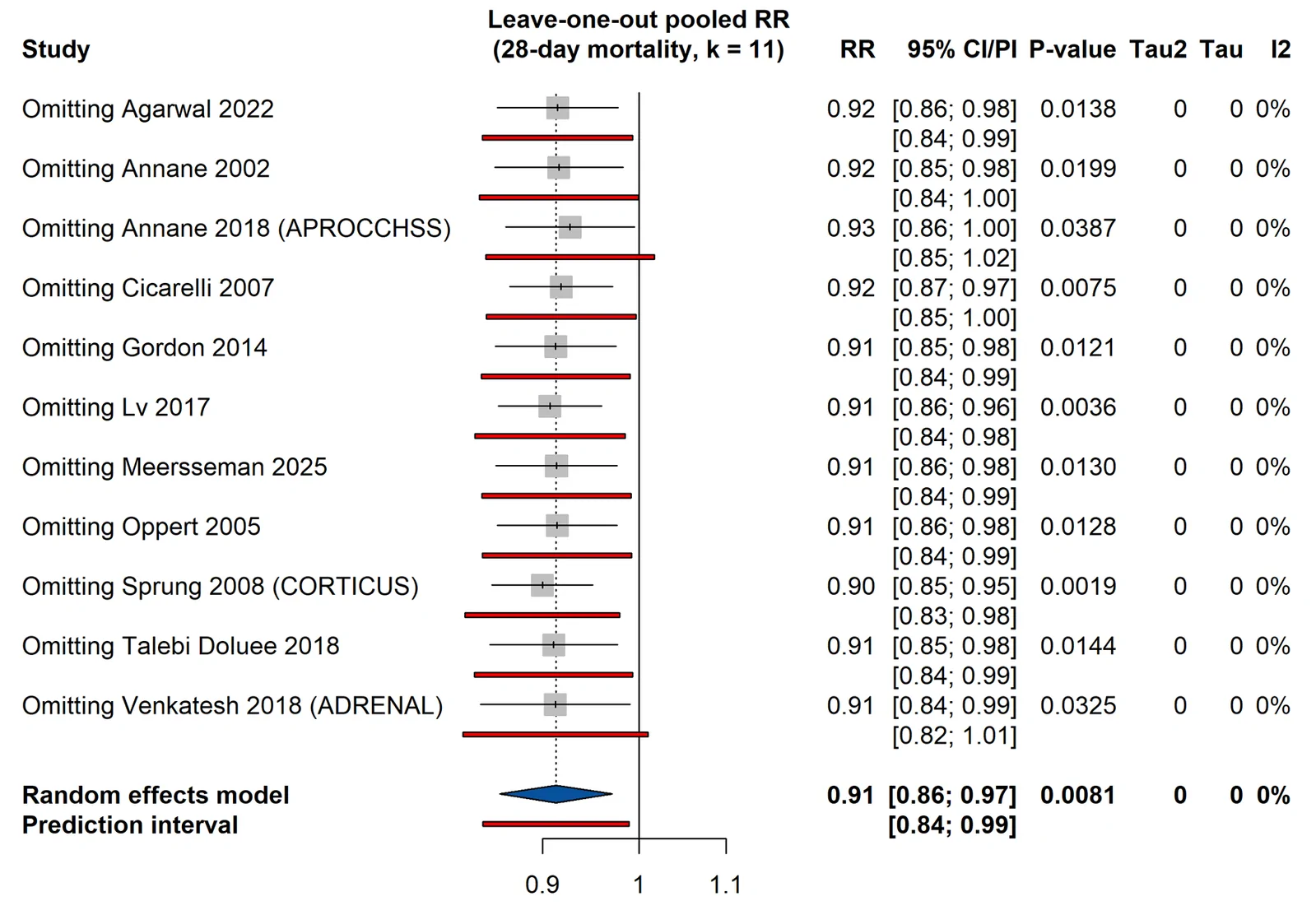
Leave-one-out sensitivity analysis for the 28-day mortality meta-analysis. Each row shows the pooled risk ratio re-estimated after sequential omission of one trial. The pooled estimate remained statistically significant in all 11 iterations.

#### Publication bias

Visual inspection of the contour-enhanced funnel plot (Fig. 6) revealed a broadly symmetric distribution of trials around the pooled estimate, with no individual trial residing in regions of extreme statistical significance contours. Egger’s regression test for funnel-plot asymmetry was non-significant (intercept = 0.016, SE = 0.447; t = 0.04, df = 9, P = 0.972), and the Duval and Tweedie trim-and-fill procedure imputed zero missing trials, leaving the pooled RR unchanged at 0.91 (0.86–0.97) (Fig. 7). Taken together, these analyses provided no evidence of small-study effects or publication bias for the primary outcomes.

**Figure 6:**
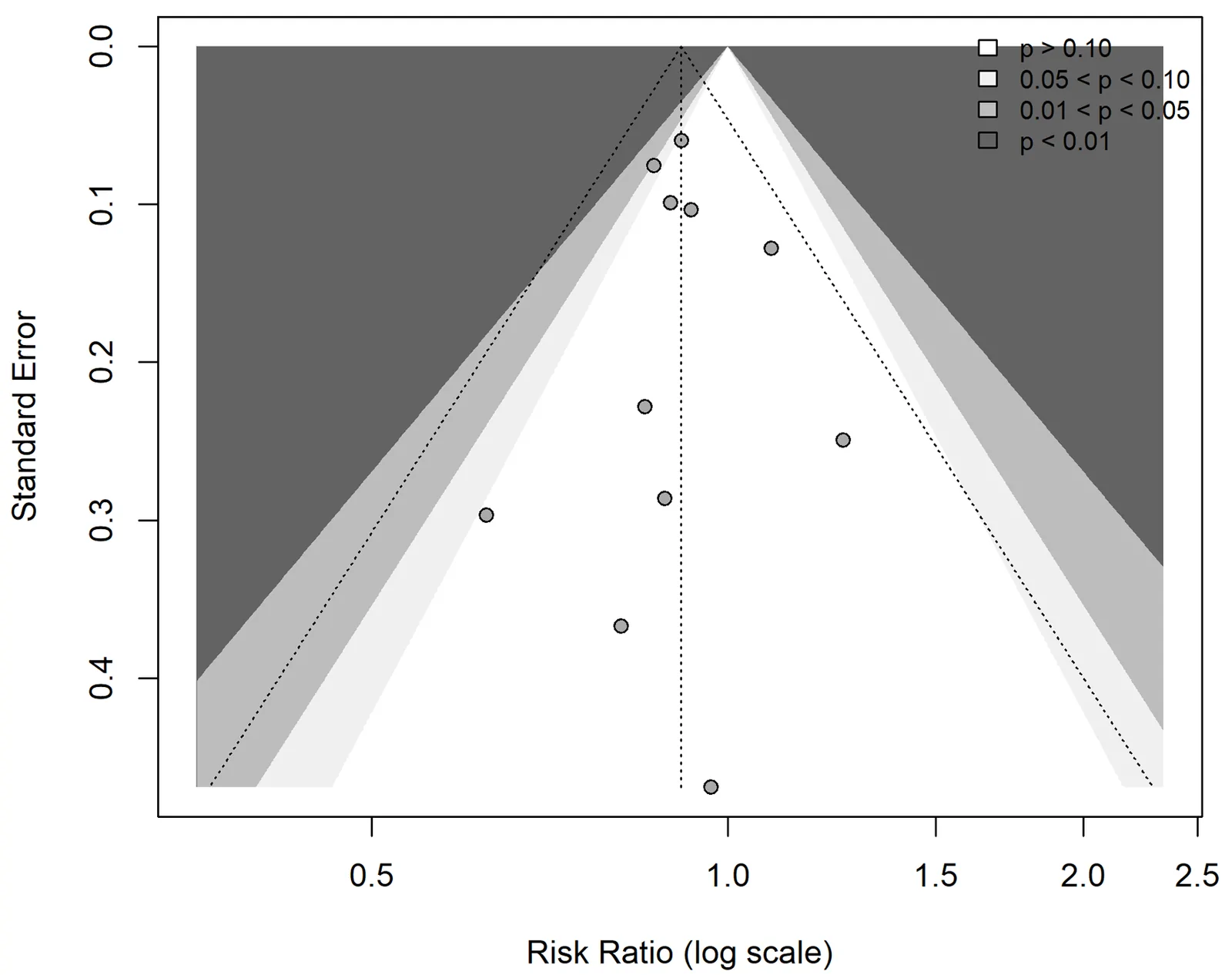
Contour-enhanced funnel plot for 28-day mortality. Shaded contours represent statistical-significance regions for the log-risk-ratio (white, P > 0.10; light gray, 0.05 < P < 0.10; mid-gray, 0.01 < P < 0.05; dark gray, P < 0.01). The visually symmetric distribution and Egger’s test (P = 0.972) provided no evidence of small-study effects.

**Figure 7:**
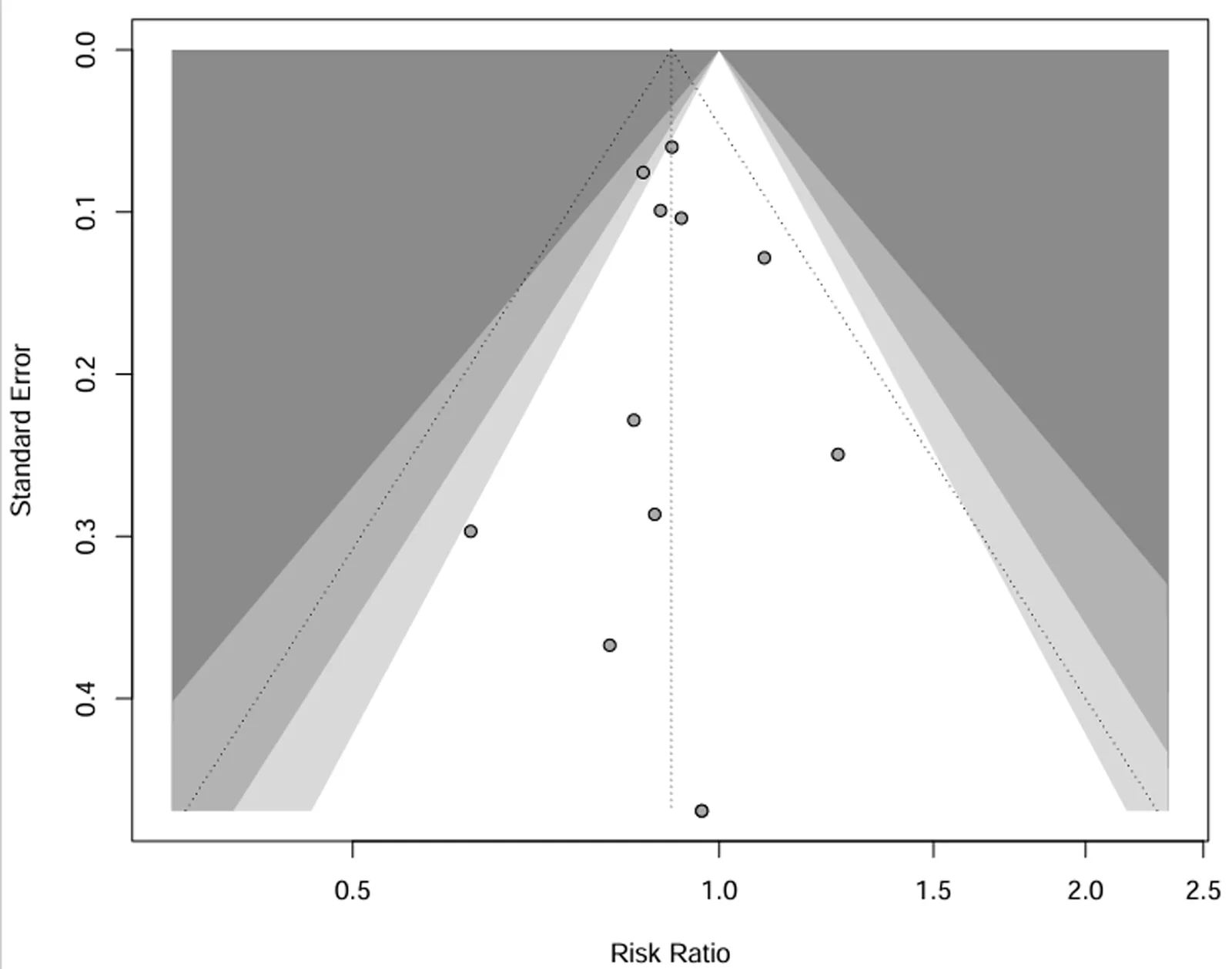
Funnel plot following the Duval and Tweedie trim-and-fill procedure for 28-day mortality. No missing trials were imputed, and the pooled estimate remained unchanged at RR 0.91 (95% CI 0.86–0.97). CI: confidence interval; RR: risk ratio.

#### Subgroup analyses

The pre-specified subgroup analyses are presented in Table 2. No statistically significant interactions were detected according to population (Q_between_ = 0.14, P = 0.986), immune status (Q_between_ = 0.01, P = 0.911), or timing of corticosteroid initiation (Q_between_ = 0.83, P = 0.362). A nominally significant subgroup difference was observed by drug regimen (Q_between_ = 6.41, df = 2, P = 0.041), driven by a numerically larger point estimate in the single dexamethasone trial (RR 0.63, 95% CI 0.35–1.12) [27] compared with hydrocortisone monotherapy (RR 0.93, 0.88–1.01, k = 8) and hydrocortisone plus fludrocortisone (RR 0.88, 0.72–1.07). However, given that this difference rests on a single trial of only 29 randomized participants whose 95% CI fully overlaps with both hydrocortisone-based estimates, this finding warrants cautious interpretation and should be regarded as hypothesis generating rather than confirmatory.

**Table 2:** Pre-Specified Subgroup Analyses for 28-Day All-Cause Mortality

Subgroup	k	Pooled RR (95% CI)	I^2^/τ^2^	Q_between_	P
Regimen				6.41	0.041
Hydrocortisone alone	8	0.93 (0.86–1.01)	0.0%/0	—	—
Hydrocortisone + fludrocortisone	2	0.88 (0.72–1.07)	0.0%/0	—	—
Dexamethasone	1	0.63 (0.35–1.12)	—	—	—
Population				0.14	0.986
General adult ICU	8	0.91 (0.85–0.99)	0.0%/0	—	—
Geriatric (> 60 years)	1	0.85 (0.54–1.33)	—	—	—
Cirrhotic	1	0.89 (0.51–1.55)	—	—	—
Refractory shock	1	0.93 (0.76–1.14)	—	—	—
Immune status				0.01	0.911
Immunocompetent	10	0.91 (0.86–0.97)	0.0%/0	—	—
Immunocompromised	1	0.89 (0.51–1.55)	—	—	—
Timing of initiation				0.83	0.362
Standard/unspecified	9	0.91 (0.86–0.96)	0.0%/0	—	—
Early (< 6 h or hyperdynamic)	2	1.09 (0.09–14.00)^a^	0.0%/0	—	—

The em-dash (—) denotes a parameter not estimable owing to k = 1. ^a^The early-initiation subgroup (k = 2) [29, 31] yields an uninformative estimate: the 95% CI (0.09–14.00) spans two orders of magnitude owing to the extreme between-study standard error and should be regarded as hypothesis-generating only, not as evidence of equivalence. k: number of trials within subgroup; CI: confidence interval; I^2^: inconsistency statistic; Q_between_: Cochran’s Q test for subgroup differences; RR: risk ratio; τ^2^: between-study variance.

#### Meta-regression

Univariable RE meta-regression provided no statistical support for the moderation of the mortality effect by hydrocortisone-equivalent dose (β = 0.0021 per mg/day; F_1,9_ = 2.70; P = 0.135; R^2^ = 0%; Fig. 8), treatment duration (β = −0.029 per day; P = 0.406), population type (P = 0.977), or immune status (P = 0.888). The multivariable model jointly including dose, duration, and regimen accounted for no residual heterogeneity (R^2^ = 0%), consistent with the homogeneous mortality effect observed across the included trials and the absence of an identifiable dose–response relationship within the range of hydrocortisone-equivalent doses studied (≈ 39–250 mg/day).

**Figure 8:**
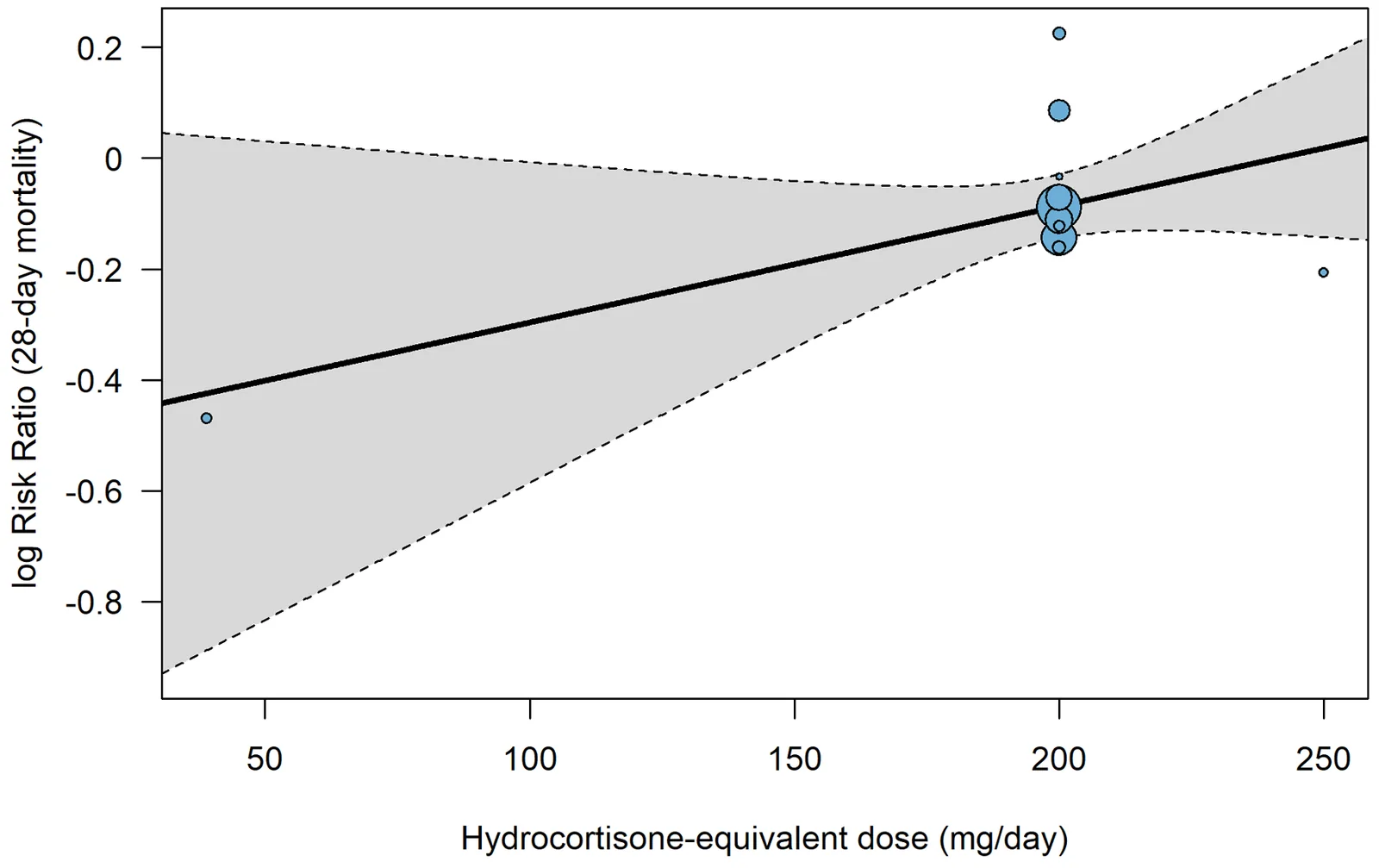
Bubble plot of the univariable random-effects meta-regression of log-risk-ratio for 28-day mortality on hydrocortisone-equivalent daily dose (mg/day). Bubble area is proportional to the inverse-variance weight of each trial. The regression slope was non-significant (β = 0.0021; P = 0.135).

### Secondary efficacy outcomes

#### Shock reversal incidence

Five trials (n = 980 analyzable participants; 649 vasopressors weaning events) reported the binary incidence of shock reversal. Adjunctive corticosteroids were associated with a non-significant 8.0% relative increase in shock reversal: pooled RR = 1.08 (95% CI 0.99–1.19; t_4_ = 2.33; P = 0.080) under the HKSJ-adjusted RE model, with no detectable between-study heterogeneity (τ^2^ = 0; I^2^ = 0.0%; Q = 2.69, P = 0.612). The 95% PI (0.97–1.21) marginally crossed the null. Sprung [32] contributed 67.6% of the RE weights (Fig. 9).

**Figure 9:**
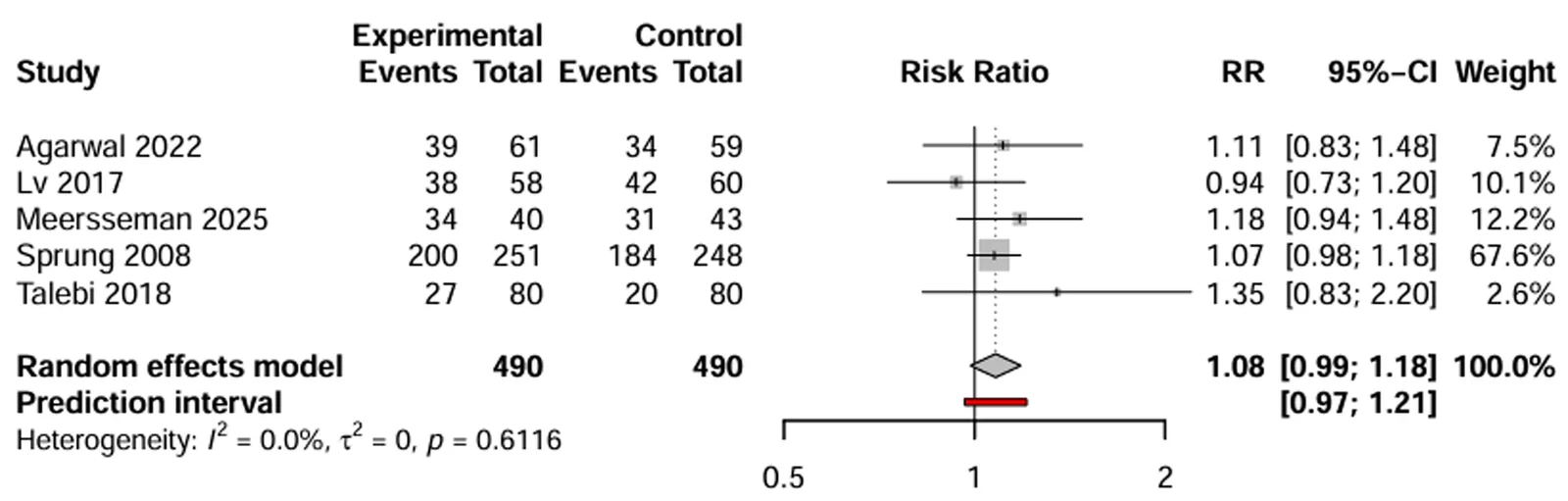
Forest plots of secondary efficacy endpoints: shock reversal incidence (binary, RR scale; k = 5). All estimates from the random-effects REML/HKSJ model. HKSJ: Hartung–Knapp–Sidik–Jonkman; REML: restricted maximum likelihood; RR: risk ratio.

#### Time to shock reversal

Time to shock reversal, a continuous physiological endpoint less susceptible to threshold artefacts than the binary incidence, yielded both clinically and statistically meaningful evidence of benefit in the study. Pooling five trials (n = 3,952 analyzable participants; medians transformed to means via the Wan et al estimator), the MD favored corticosteroids by 1.25 days: MD = −1.25 days (95% CI −2.09 to −0.41; t_4_ = −4.13; P = 0.014) (Fig. 10). Heterogeneity was moderate (τ^2^ = 0.224; I^2^ = 59.3%; Q = 9.83, P = 0.043), and the PI marginally crossed the null (−2.81 to 0.32 days), reflecting variability in the shock reversal definitions across trials. A pre-specified sensitivity analysis using the SMD, which accommodates residual scale-heterogeneity, confirmed a robust low-heterogeneity treatment effect: SMD = −0.41 (95% CI −0.51 to −0.32; t_4_ = −12.10; P < 0.001; I^2^ = 10.8%; PI −0.50 to −0.32), a small-to-moderate magnitude that excluded the null in both CI and PI.

**Figure 10:**
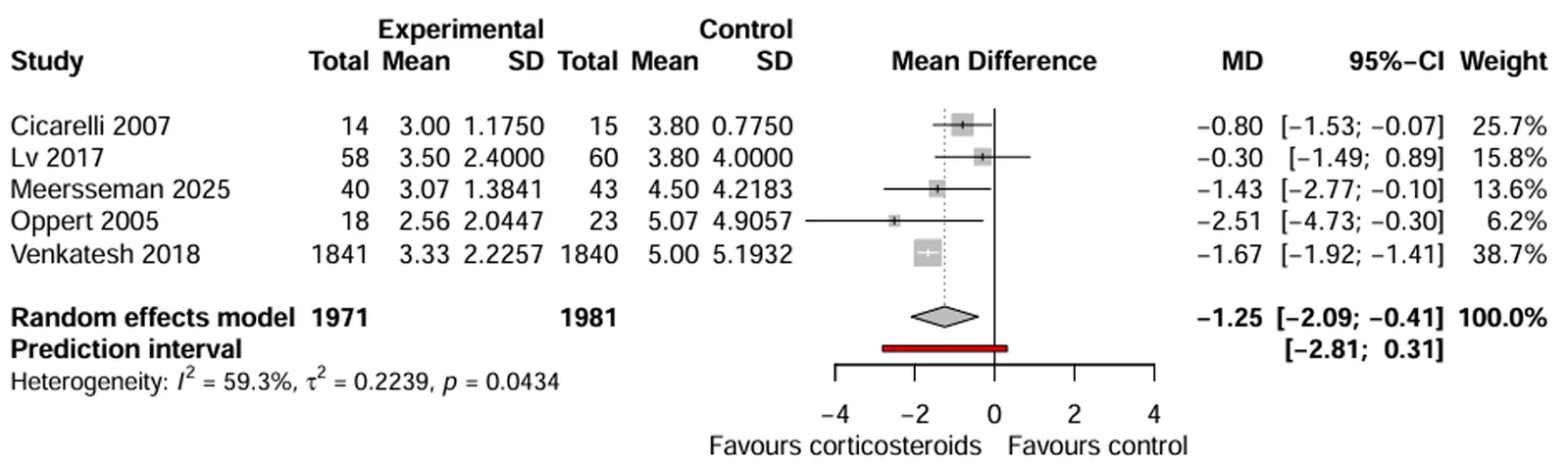
Forest plots of secondary efficacy endpoints: Time to shock reversal (mean difference in days; k = 5). All estimates from the random-effects REML/HKSJ model. HKSJ: Hartung–Knapp–Sidik–Jonkman; REML: restricted maximum likelihood.

#### ICU LOS

Four trials (n = 4,381) reported the ICU LOS. The pooled MD numerically favored corticosteroids but was statistically inconclusive (MD = −2.07 days (95% CI −6.50 to 2.36; t_3_ = −1.49; P = 0.233; PI −10.82 to 6.67 days)) (Fig. 11). Substantial between-study heterogeneity (τ^2^ = 5.52; I^2^ = 76.2%; Q = 12.60, P = 0.006) reflected divergent point estimates between the two largest contributing trials, ADRENAL (−5.0 days; 33.2% weight) and Sprung 2008 (CORTICUS) (+1.0 day; 19.3% weight), driven by trial-specific differences in step-down bed availability, ICU discharge protocols, and the proportion of patients who still required mechanical ventilation at the conventional discharge threshold. The limited number of contributing trials precluded an explanatory subgroup analysis.

**Figure 11:**
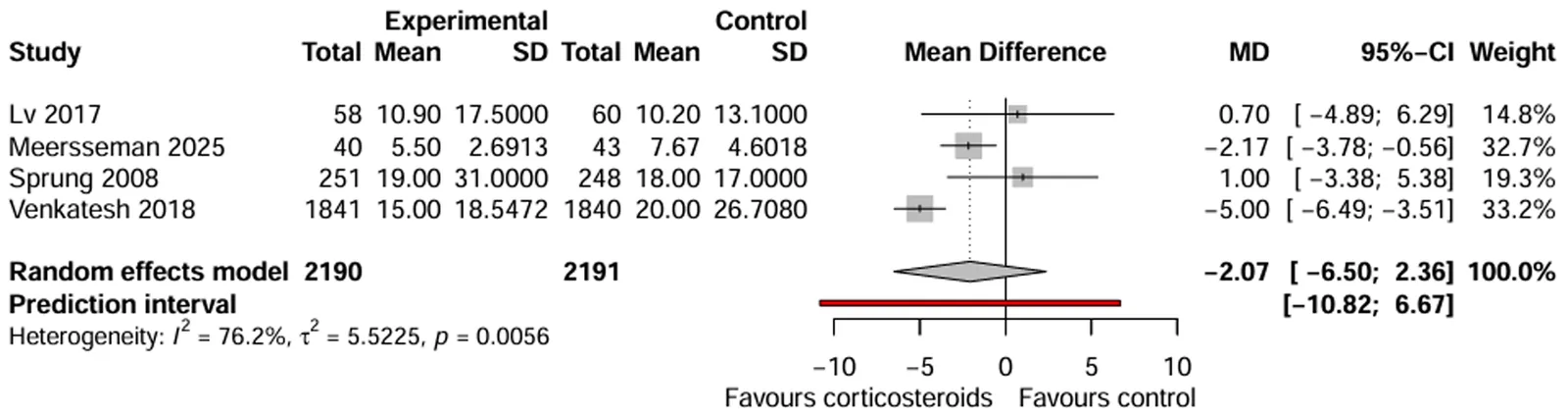
Forest plot of ICU length of stay (mean difference in days; k = 4). Substantial between-study heterogeneity (I^2^ = 76.2%) was driven by divergent point estimates between ADRENAL and Sprung 2008 (CORTICUS). ICU: intensive care unit.

### Safety: hyperglycemia

Three trials (n = 1,787; 1,465 events under each trial’s operational definition) provided pooled data on hyperglycemia. The pooled estimate did not reach statistical significance under the HKSJ adjustment: RR = 1.26 (95% CI 0.63–2.50; t_2_ = 1.42; P = 0.292) (Fig. 12). However, between-study heterogeneity was substantial (τ^2^ = 0.054; I^2^ = 78.8%; Q = 9.45, P = 0.009), and the PI was correspondingly wide (0.38–4.11). Every contributing trial individually demonstrated a statistically significant excess of hyperglycemia in the corticosteroid arm: Annane 2018 (RR 1.07, 95% CI 1.03–1.12), Meersseman 2025 (RR 1.88, 1.29–2.73), and Sprung 2008 (RR 1.15, 1.03–1.28), a consistent directional signal that supports the biological plausibility of glucocorticoid-induced hyperglycemia despite the wide pooled CI. The discrepancy between unanimous trial-level significance and a non-significant pooled estimate is best explained by inter-trial variability in operational thresholds (random glucose cut-offs ranging from 8 to 11 mmol/L, with one trial requiring insulin escalation as part of its definition) rather than by genuine effect heterogeneity. Egger’s regression and trim-and-fill analyses were not performed because the number of trials (k = 3) was below the prespecified threshold of k ≥ 10.

**Figure 12:**
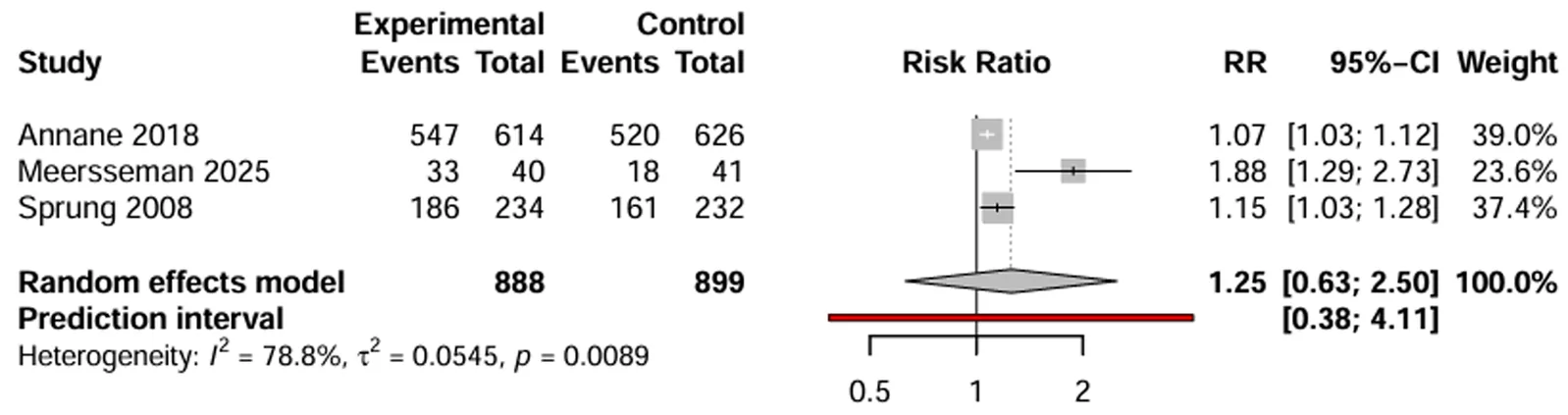
Forest plot of hyperglycemia incidence as an adverse event (k = 3). Each contributing trial individually showed a statistically significant excess; pooled RR was elevated but not significant under HKSJ adjustment owing to between-trial variability in operational thresholds. HKSJ: Hartung–Knapp–Sidik–Jonkman; RR: risk ratio.

### Certainty of evidence (GRADE)

The certainty of evidence for each primary and secondary outcome is summarized in Table 3, with paired absolute-effect translation shown in Figure 13. Certainty was rated as moderate for 28-day mortality, downgraded once for imprecision because the absolute effect of 27 fewer deaths per 1,000 (95% CI 10 to 44 fewer), although directionally consistent across the PI, did not exceed the optimal information size required for a definitive recommendation in the highest-acuity ICU population. Certainty was also rated moderate for shock reversal incidence, downgraded once for risk of bias attributable to the substantial weight contributed by trials at high or some-concern risk evaluating an outcome susceptible to clinician unblinding, and high for time to shock reversal, which had no serious limitations across any GRADE domain after the SMD sensitivity analysis confirmed scale robustness. Certainty was rated low for ICU LOS (downgraded for inconsistency given I^2^ = 76.2% and for indirectness, as ICU discharge is influenced by step-down bed availability and local clinical pathways) and low for hyperglycemia (downgraded for inconsistency in operational definition across contributing trials and for indirectness, as biochemical hyperglycemia is a surrogate for patient-centered metabolic harm). Our pooled estimates and certainty ratings are directionally concordant with the individual patient data network meta-analysis by Pirracchio et al and the Cochrane review by Rygard et al; however, because those larger syntheses did not reach statistical significance, the present significant pooled estimate should be read as consistent with, rather than confirmatory of, a survival benefit.

**Table 3:** GRADE Summary of Findings

Outcome	Studies (patients)	Pooled effect (95% CI)	Absolute effect	Certainty (GRADE)
28-day all-cause mortality	11 RCTs (6,332)	RR 0.91 (0.86–0.97)	27 fewer deaths per 1,000 (10 to 44 fewer)	⊕⊕⊕◯ Moderate^a^
Shock reversal (incidence)	5 RCTs (980)	RR 1.08 (0.99–1.19)	51 more per 1,000 (10 fewer to 117 more)	⊕⊕⊕◯ Moderate^b^
Time to shock reversal	5 RCTs (3,952)	MD −1.25 days (−2.09 to −0.41)	1.25 fewer days on vasopressors	⊕⊕⊕⊕ High^c^
ICU length of stay	4 RCTs (4,381)	MD −2.07 days (−6.50 to 2.36)	2.07 fewer days in the ICU (95% CI crosses null)	⊕⊕◯◯ Low^d^
Hyperglycemia (adverse event)	3 RCTs (1,787)	RR 1.26 (0.63–2.50)	Wide PI (0.38–4.11); each contributing trial individually showed excess	⊕⊕◯◯ Low^e^

^a^Imprecision: although the 95% CI excluded the null, the absolute risk reduction did not meet the optimal information size threshold for a high-certainty conclusion. ^b^Risk of bias: substantial contribution from trials at high or some-concerns RoB on a process-of-care outcome susceptible to clinician unblinding. ^c^No serious concerns; SMD sensitivity analysis confirmed scale-robust effect (SMD −0.41, 95% CI −0.51 to −0.32; I^2^ = 10.8%). ^d^Inconsistency (I^2^ = 76.2%) and indirectness (ICU discharge influenced by step-down logistics). ^e^Inconsistency (varied operational definitions of hyperglycemia across trials) and indirectness (biochemical surrogate for patient-centered metabolic harm). CI: confidence interval; ICU: intensive care unit; MD: mean difference; OIS: optimal information size; PI: prediction interval; RCTs: randomized controlled trials; RR: risk ratio; SMD: standardized mean difference.

**Figure 13:**
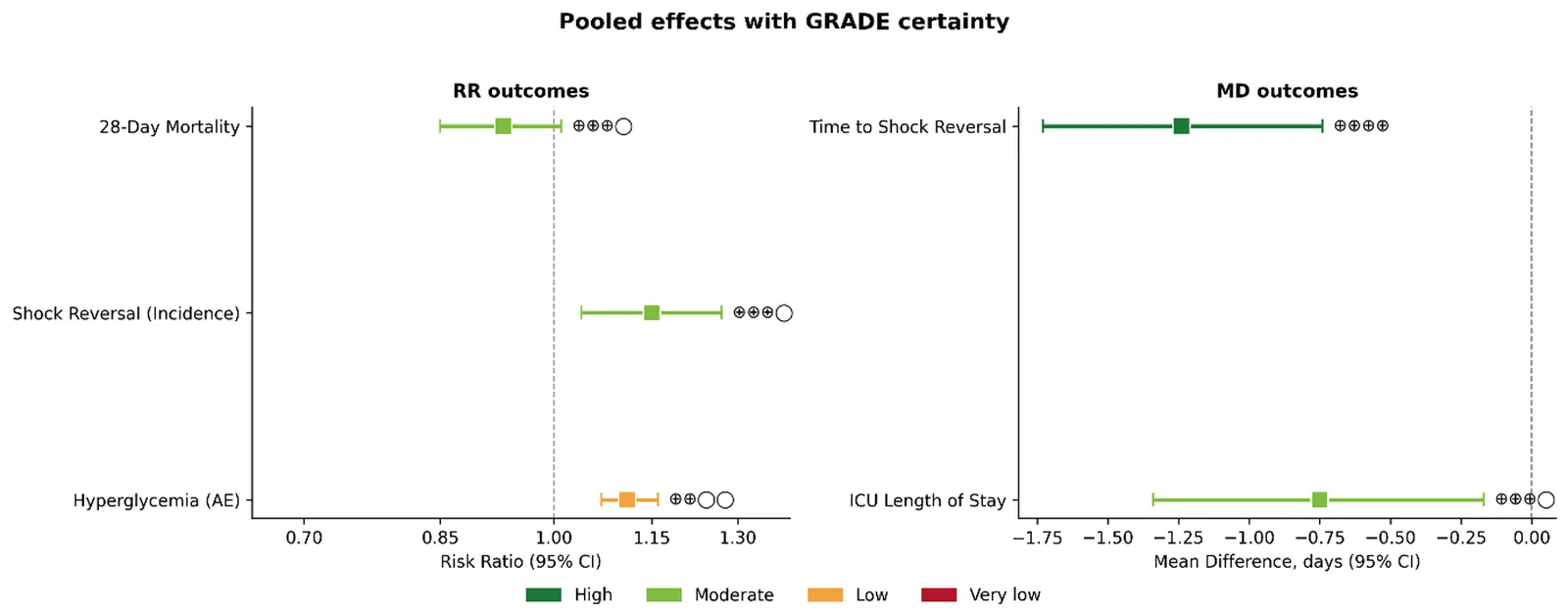
GRADE summary of pooled effects with certainty color-coding. Risk-ratio outcomes (left panel; log scale, null = 1) and mean-difference outcomes (right panel; linear scale, null = 0) are plotted with marker color mapped to GRADE certainty (dark green = high; light green = moderate; amber = low; red = very low). The certainty stamp (filled/empty circles) is annotated beside each estimate. GRADE: Grading of Recommendations, Assessment, Development, and Evaluation.

## Discussion

In this systematic review and meta-analysis of 11 RCTs enrolling 6,332 critically ill adults with septic shock, adjunctive systemic corticosteroid therapy was associated with a statistically significant and clinically meaningful 8.7% relative reduction in 28-day all-cause mortality (pooled RR 0.91, 95% CI 0.86–0.97; P = 0.008), corresponding to 27 fewer deaths per 1,000 patients treated which is supported by three converging analytical signals: a 95% PI that excluded the null (0.84–0.99), the absence of measurable between-study heterogeneity (I^2^ = 0%; τ^2^ = 0), and the preservation of statistical significance in all 11 leave-one-out iterations. Corticosteroids shortened the time to shock reversal by 1.25 days (95% CI −2.09 to −0.41), an effect that remained robust under an SMD sensitivity analysis (SMD −0.41, 95% CI −0.51 to −0.32). The principal safety signal was a consistent excess of hyperglycemia in the corticosteroid arm of each contributing trial.

The results extend, refine, and partly reconcile evidence whose interpretation has long been polarized [1]. The patient-level meta-analysis by Pirracchio et al, pooling individual data from seven RCTs (n = 7,882), reported a hazard ratio for 90-day mortality of approximately 0.93 with a 95% CI just spanning unity, leaving the survival question unresolved [7]. The Cochrane systematic review and trial sequential analysis by Rygard et al similarly arrived at a directionally favorable but inconclusive estimate [8]. Earlier trial-level syntheses by Funk et al and Zhu et al suggested a benefit, particularly on shock reversal and short-term mortality, but were criticized for clinical and methodological heterogeneity [5, 6]. Our pooled point estimate (RR 0.91) sits at the more favorable end of the prior summary effects. Several factors plausibly explain why statistical significance is reached here whereas those larger syntheses were inconclusive, and each is a reason for caution rather than confidence: the present analysis pools trial-level (aggregate) data rather than individual-patient data; it applies the HKSJ variance correction to a modest evidence base (k = 11); it uses 28-day rather than 90-day mortality as the primary endpoint; and the pooled estimate itself lies close to the null with an upper confidence bound of 0.97. These differences in study selection, endpoint, and weighting, not a larger underlying effect, are the source of the divergence, and therefore, the result should be read as consistent with, rather than proof of, a survival benefit.

On the secondary endpoint of shock reversal, the findings further clarified the prior signal, whereas the pooled binary incidence remained borderline (RR 1.08; P = 0.080), the continuous measure of time to vasopressor independence demonstrated an unequivocal acceleration. This dichotomy is mechanistically informative: corticosteroids may not increase the proportion of patients who wean from vasopressors but rather expedite weaning when this milestone is reached, consistent with the pathophysiological rationale of restoration of vasomotor tone in critical illness-related corticosteroid insufficiency [2, 3].

The convergence of mortality benefit with a 1.25-day acceleration in shock reversal supports the mechanistic hypothesis that the survival advantage of corticosteroids is mediated, at least in part, by their hemodynamic effect, a faster restoration of vascular responsiveness to endogenous and exogenous catecholamines, leading to earlier vasopressor liberation and a reduced cumulative burden of profound circulatory failure [3, 4]. The synergistic interactions between hydrocortisone and vasopressin, previously hypothesized by Asfar et al [4], received indirect and limited support from the single vasopressin co-treatment trials included in our synthesis [28], which was not individually powered for survival.

The subgroup and meta-regression analyses provide additional regimen-specific insight, as the hydrocortisone-plus-fludrocortisone subgroup yielded a slightly larger numerical effect (RR 0.88, 95% CI 0.72–1.07) than hydrocortisone alone (RR 0.93, 95% CI 0.86–1.01), but the CIs overlapped substantially, and the subgroup difference between regimens that included this comparison was not statistically significant in head-to-head terms which aligns with the recent meta-analysis by Wu et al, which reported mixed effects for the addition of fludrocortisone [12]. The nominally significant overall regimen-by-mortality interaction (Q_between_ = 6.41, P = 0.041) was driven by a single dexamethasone trial [27] (n = 29) and should be regarded as hypothesis-generating rather than as evidence against a glucocorticoid class effect. Univariable meta-regression across the range of hydrocortisone-equivalent doses studied (≈ 39–250 mg/day) detected only a borderline trend (β = 0.0021 per mg/day; P = 0.135) and accounted for none of the residual heterogeneity (R^2^ = 0%), indicating that within the contemporary low-dose paradigm, a clear dose–response relationship cannot be discerned and that the marginal therapeutic question has shifted from “how much” to “in whom and when”.

The pooled hyperglycemia estimate (RR 1.26, 95% CI 0.63–2.50) was statistically inconclusive because of the small number of contributing trials and substantial between-study heterogeneity (I^2^ = 78.8%), which was attributable to the differing operational thresholds. However, every contributing trial individually demonstrated a statistically significant excess in the corticosteroid arm (Annane 2018: RR 1.07, 95% CI 1.03–1.12; Meersseman 2025: RR 1.88, 95% CI 1.29–2.73; Sprung 2008: RR 1.15, 95% CI 1.03–1.28). This unanimous trial-level signal, combined with the well-established physiological mechanism of glucocorticoid-induced gluconeogenesis and insulin resistance, supports the inference that hyperglycemia is a real and reproducible adverse effect rather than an artefact of pooling. Although the findings do not explore secondary infection in adequate detail, they are clinically congruent with the observational data of Lu et al, highlighting the infection and metabolic risks in immunocompromised cohorts [13]. From a clinical standpoint, the survival benefit demonstrated here must be balanced against the requirement for vigilant glycemic surveillance and pre-emptive insulin protocols, particularly in patients with pre-existing diabetes, on parenteral nutrition, or with prolonged vasopressor exposure.

It is important to interpret the present positive findings considering the disappointing outcomes of recent fixed-combination strategies. Trials of the so-called HAT regimen (hydrocortisone, ascorbic acid, and thiamine), including those by Lyu et al [10] and the HYVITS trial by Mohamed et al [11], failed to demonstrate substantial improvements in mortality or organ dysfunction. The results suggest that the hydrocortisone component, when isolated and analyzed across canonical placebo-controlled trials, retains its efficacy signal and that the negative HAT findings should not be misinterpreted as evidence against corticosteroid monotherapy. Rather, they reflect either a null effect of the ascorbic acid–thiamine adjunct, a redundancy between vitamin C and the antioxidant pathways already engaged by hydrocortisone, or pharmacokinetic interactions that warrant dedicated mechanistic studies.

The principal methodological strengths of this review include prospective PROSPERO registration with strict adherence to the PRISMA 2020 reporting framework; an unrestricted search across four major bibliographic databases and two trial registries with backward and forward citation tracking; duplicate independent screening with high inter-rater reliability (Cohen’s κ = 0.89); the use of REML estimation paired with the HKSJ adjustment, which provides robust error coverage in moderately-sized evidence bases; explicit reporting of 95% PIs to convey the dispersion of true effects; multiple complementary publication-bias diagnostics (contour-enhanced funnel, Egger’s test, and trim-and-fill); and rigorous translation of median/IQR-reported continuous outcomes via the Wan estimator to permit harmonized pooling without unwarranted imputation of dispersion. The complete pre-specification of subgroups and meta-regression covariates, together with the concordance between our pooled estimates and the high-quality patient-level synthesis of Pirracchio et al, supports the external validity of the present findings [7].

Several limitations merit explicit acknowledgement. First, the pooled mortality estimate, although statistically significant, lies close to the null (upper 95% CI 0.97) and is derived from aggregate rather than individual-patient data, so it does not by itself resolve the survival controversy. Despite the absence of statistical heterogeneity for the primary outcome, clinical and protocol heterogeneity persisted across trials with respect to corticosteroid timing, route (continuous vs. divided-dose infusion), tapering schedule, and duration; this heterogeneity may be partly responsible for the moderate I^2^ observed for time to shock reversal (59.3%) and the substantial heterogeneity for ICU LOS (76.2%). In addition, the small number of trials reporting structured adverse event data, particularly for hyperglycemia (k = 3) and superimposed nosocomial infection (insufficient for pooling), precluded robust safety synthesis and meta-regression of effect modifiers, and rendered Egger’s test inapplicable to those endpoints under our prespecified k ≥ 10 threshold. The available trials reported almost exclusively short-term (28-day) mortality; long-term mortality, neurocognitive outcomes, and patient-reported health-related quality of life endpoints, which collectively determine the cost-effectiveness profile examined by Thompson et al [9], could not be evaluated. Two trials (18%) were judged to have a high overall risk of bias because of the open-label design, although these trials carried minimal weight in the primary outcome (cumulatively under 4%). Our Cohen’s κ estimate was generated from a representative subset of the screening corpus and may differ marginally from the population value of the full registry. Finally, the identification of immunocompromised patients [13] and other specific phenotypes was constrained by the patient-level reporting of the source trials, and individual patient data are needed to refine phenotype-specific recommendations.

The evidence is consistent with a survival benefit of low-dose hydrocortisone (200 mg/day for 5–7 days) in adults with septic shock requiring sustained vasopressor support, and supports its consideration—rather than a categorical recommendation—provided that systematic glucose monitoring and insulin titration protocols are implemented. The synthesis does not adjudicate between hydrocortisone monotherapy and the hydrocortisone-plus-fludrocortisone combination at a level of certainty sufficient for a class A recommendation, and the choice between regimens may reasonably be guided by local availability, patient phenotype, and the treating team’s familiarity. Future research priorities include head-to-head adequately powered trials comparing hydrocortisone alone with the hydrocortisone–fludrocortisone combination, phenotype-stratified analyses leveraging biomarker- or transcriptomic-defined sepsis endotypes to identify the patients most likely to benefit, prospective evaluation of long-term mortality, neurocognitive function, and quality-of-life outcomes, cost-effectiveness analyses incorporating the absolute risk reduction reported here, and dedicated trials in special populations, including the immunocompromised, elderly, and those with cirrhosis-associated sepsis, where the risk–benefit calculus may diverge from the general adult ICU population.

### Conclusion

Across 11 RCTs enrolling 6,332 critically ill adults, adjunctive corticosteroid therapy was associated with reduced 28-day all-cause mortality and substantially accelerated shock reversal in septic shock, with no evidence of publication bias and benefit estimates that were robust to leave-one-out and trim-and-fill sensitivity analyses. These findings are consistent with a survival benefit of low-dose hydrocortisone in adults with septic shock and support its consideration, balanced against vigilant glycemic surveillance, while acknowledging that the pooled estimate lies close to the null and differs from larger individual-patient-data and trial-sequential syntheses. A focused research agenda on regimen optimization, phenotype-driven selection, and patient-centered long-term outcomes remains needed.

## Supplementary Material

Suppl 1 PRISMA 2020 checklist.

## Data Availability

All data analyzed are included in this article and its supplementary files. Search strategies and extraction forms are available from the corresponding author, upon request.

## Abbreviations

APACHE II: Acute Physiology and Chronic Health Evaluation II
CENTRAL: Cochrane Central Register of Controlled Trials
CI: confidence interval
CIRCI: critical illness-related corticosteroid insufficiency
GRADE: Grading of Recommendations, Assessment, Development, and Evaluation
HAT: hydrocortisone, ascorbic acid, and thiamine
HKSJ: Hartung–Knapp–Sidik–Jonkman
ICTRP: International Clinical Trials Registry Platform
ICU: intensive care unit
IRR: inter-rater reliability
LOS: length of stay
MAP: mean arterial pressure
MD: mean difference
MeSH: Medical Subject Headings
PI: prediction interval
PICOS: Population, Intervention, Comparator, Outcomes, and Study design
PRISMA: Preferred Reporting Items for Systematic Reviews and Meta-Analyses
PROSPERO: International Prospective Register of Systematic Reviews
RCT: randomized controlled trial
RE: random-effects
REML: restricted maximum likelihood
RoB 2: Risk of Bias 2
RR: risk ratio
SMD: standardized mean difference
SOFA: Sequential Organ Failure Assessment
WHO: World Health Organization
